# Supplementary material for: Comprehensive Annotation and Functional Exploration of MicroRNAs in Lettuce
Source: Front Plant Sci. 2021 Dec 24;12:781836. doi: 10.3389/fpls.2021.781836 (PMC8739914; doi:10.3389/fpls.2021.781836)
Supplement: Supplementary file 2 [file Data_Sheet_8.ZIP › GSM2230750.plot/Lsa-miRN1652a_Lsat_1_v5_gn_7_48101.1_38_TPlot.pdf]

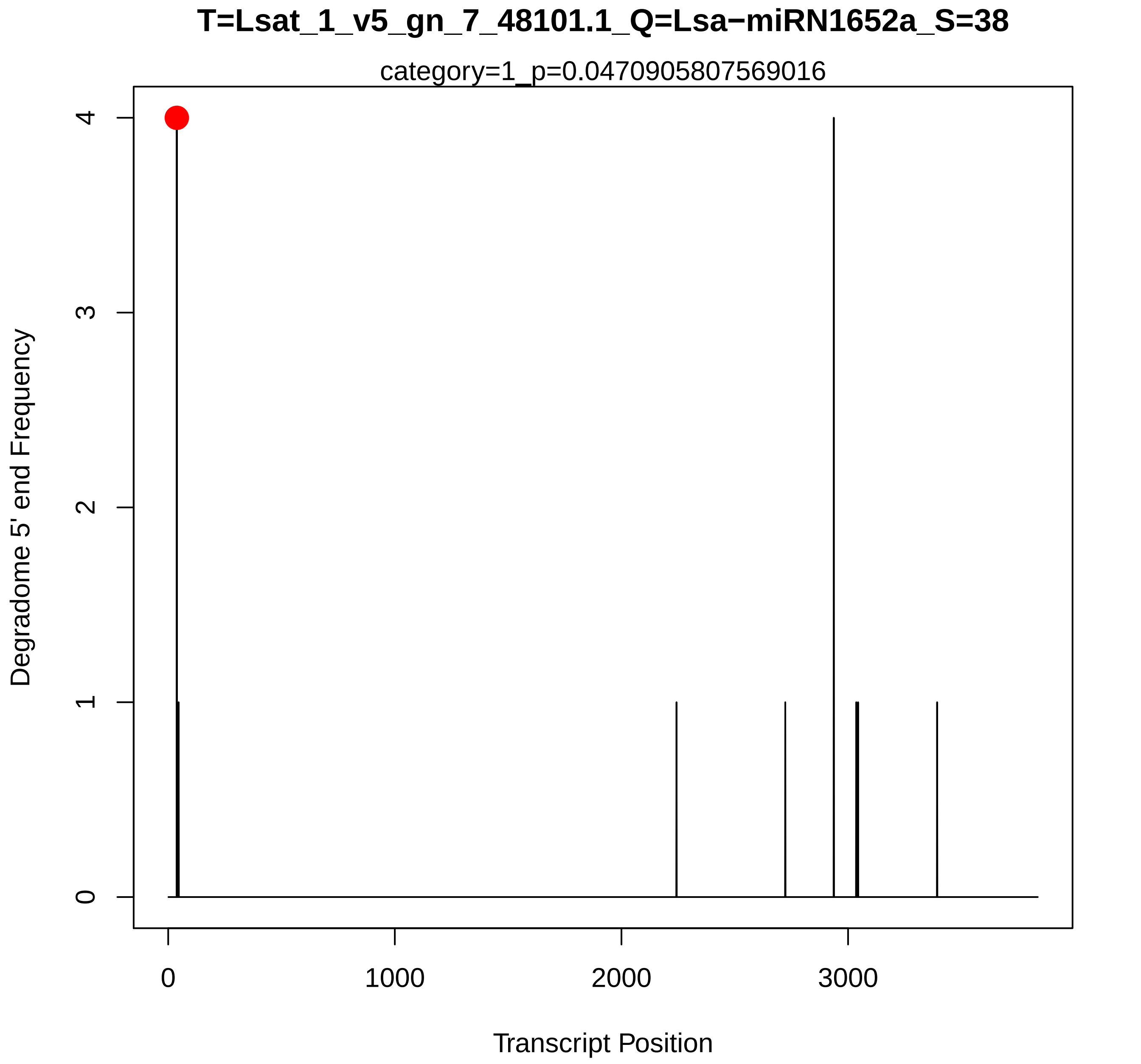
T=Lsat_1_v5_gn_7_48101.1_Q=Lsa−miRN1652a_S=38
category=1_p=0.0470905807569016
Degradome 5' end Frequency
0
1
2
3
4
0
1000
2000
3000
Transcript Position
